# Lever-Test Accuracy in Detecting Anterior Cruciate Ligament (ACL) Tears: A Single Institution Experience

**DOI:** 10.7759/cureus.46313

**Published:** 2023-10-01

**Authors:** Mohammed Y Sarhan, Alaa Akel, Basel Balbisi, Yesar Faouri, Lama Alfraihat, Bashar I naser, Mohammad Abu-Jeyyab

**Affiliations:** 1 Orthopedic Surgery, Hashemite University, Al-Zarqaa, JOR; 2 Orthopaedic Surgery, School of Medicine, Mutah University, Al-Karak, JOR; 3 Orthopaedics, Hashemite University, Al-Zarqaa, JOR; 4 Orthopaedics, Prince Hamzah Hospital, Amman, JOR; 5 Special Surgery, School of Medicine, Mutah University, Amman, JOR

**Keywords:** prospective studies, supine position, reliability, magnetic resonance imaging, lachman test, lever test, anterior cruciate ligament injuries, anterior cruciate ligament

## Abstract

Background

One of the most often damaged ligaments in the knee is the anterior cruciate ligament (ACL). With the increased occurrence of ACL injuries, there is a greater need for clinical diagnostics to rule in or rule out a suspected rupture. The Lever Test, a novel clinical tool for diagnosing ACL rupture, has been presented, with preliminary trials indicating encouraging results.

Methods

This is a prospective, blinded, diagnostic accuracy study. The aim of this study was to evaluate the accuracy of the Lever Test and other common clinical tests (Anterior Drawer Test, Lachman Test, Pivot Shift Test) for diagnosing ACL injuries. The study enrolled 23 patients who had knee pain, instability, and locking symptoms. The clinical tests were performed on the patients in supine position before, during, and after anesthesia. The results of the clinical tests were compared with MRI findings to determine the sensitivity of each test.

Results

The patients consisted of 17 men and six women, with a mean age of 30.4±9.95 years. 18 patients had complete tears, four had partial tears, and one had intact ACL damage. 10 (44%) complained in the right knee, 13 (56%) in the left knee, and two (9%) had a generalized ligamentous laxity. 21 (91%) complained of giving away, 22 (96%) complained of knee pain, and 10 (43%) complained of locking of the knee. On the ipsilateral leg examination, pre-operative positivity of Lever Sign was 44%, Lachman 83%, and Anterior Drawer 67%. After being given anesthesia, test positivity rates were 44% for Lever Sign and 56% for Pivot Shift. Post-operative positivity of Lever Sign was 17%, Lachman 39%, and Anterior Drawer 35%. Mcnemar test p values were significant for the difference of positivity anterior drawer test (p=0.002) and were not significant on Lever Sign (p=0.7) and Lachman tests (p=0.13). Correlation analysis showed a weak but not statistically significant interrater reliability between MRI findings and Lever Sign (p=0.846) (Kappa= 0.2). On the contralateral leg examination, the pre-operative positivity of Lever Sign was 9%, Lachman 17%, and Anterior Drawer 22%

Conclusion

The study suggests that the Lever Test has lower accuracy than other clinical tests when comparing the results of tests with MRI findings. As a result, Lever Test should be used in combination with other clinical tests to accurately rule out suspected ACL injuries.

## Introduction

The anterior cruciate ligament (ACL) is one of the four primary ligaments of the knee joint. It performs the role of keeping the tibia from slipping forward on the femur. ACL tears are a common injury, particularly in sports involving abrupt pauses and changes in direction, such as football, basketball, and hockey [1].

An ACL injury is frequently diagnosed clinically, depending on the patient's history and physical examination. Imaging procedures, such as MRI, can assist in confirming the diagnosis and rule out other potential ailments [2].

The Lever Sign Test is a clinical examination that has been characterized as a feasible alternative to MRI for the diagnosis of ACL injuries [2]. The patient is asked to lie on their back with their knee bent at a 90-degree angle for the test. The examiner then pushes down on the patient's foot while applying a valgus force to the knee. The test is positive for an ACL injury if the tibia moves forward on the femur [3].

It is crucial to remember, however, that the Lever Sign Test is not flawless. The test has a 13% false-positive rate. This suggests that 13% of patients who have a negative Lever Sign Test have an ACL injury [4]. As a result, the Lever Sign Test should not be used only to diagnose ACL rupture. It should be used in combination with other clinical tests like the Lachman Test and Pivot Shift Test, as well as imaging procedures like MRI.

Overall, the Lever Sign Test appears to be a good clinical tool for detecting ACL injuries. The test is simple to administer and has high sensitivity and specificity. The test is not ideal, however, and it should not be utilized as the primary diagnostic tool for ACL rupture. It should be employed with other clinical testing and imaging examinations.

## Materials and methods

Study design

The purpose of this prospective blinded diagnostic accuracy research study was to assess the accuracy of the Lever Test in detecting ACL tears in individuals with knee injuries.

Study setting and population

Between November 2021 and November 2022, the study was conducted in a tertiary care facility. The study included 23 individuals who attended the emergency room or orthopedic clinic with acute or persistent knee pain and a suspected ACL tear. Aged between 18 and 65 years, ability to execute the Lever Test, and willingness to undergo MRI of the knee were the inclusion criteria. Previous knee surgery, MRI contraindications, and incapacity to offer informed permission were all exclusion criteria. The study was accepted by the ethics committee of Prince Hamza Hospital under the number PH8985. Every single participant signed a detailed consent form in Arabic.

Data collection

Each record's data was obtained using a standardized form that comprised the following variables:

Timestamp - Specific Patient Identifier Number - Age of Patient - Place of Assessment - Date of Assessment - Side of Main Complaint - Generalized Ligamentous Laxity - Symptoms - Anterior Drawer Test - Lachman Test - Pivot Shift Test (under Anesthesia) - Lever Sign - Lever Sign (under Anesthesia) - MRI - ACL - MRI - Other Findings

The procedure of performing the Lever Test

A senior expert-trained examiner who was blinded to the MRI data completed the Lever Test. The patient was positioned supine on the examination table, with both knees flexed at 90 degrees and both feet flat on the table. The examiner next pulled both heels off the table to see if one tibia sagged posteriorly more than the other, indicating a positive ACL tear test.

Imaging tests

The MRI of the knee was conducted by a radiologist who was unaware of the results of the Lever Test. The MRI was considered the gold standard for determining ACL tears. The presence or absence of total or partial ligament rupture was used to classify the MRI results as positive or negative for ACL injury.

Outcome measures

The primary result of the study was the sensitivity of the Lever Test in comparison with other classical tests (Anterior Drawer Test, Lachman, Pivot Shift Test) in identifying ACL. The secondary result of the study was to determine the degree of agreement between the Lever Test and MRI findings.

Patient confidentiality and ethical considerations

In performing human research, this study adhered to the ethical values of respect for people, beneficence, and fairness. The study protocol was approved by the hospital's institutional review board (IRB) and followed the Helsinki Declaration. All subjects provided informed permission before being enrolled in the research. The goal, methods, dangers, rewards, and voluntary nature of the study were all explained to the participants. They were also promised that they might leave the study at any moment without penalty.

The study also guaranteed that the data of the participants were kept personal and private. The information was gathered using a standardized form that solely provided number IDs and no personal information. The information was kept in a secure database that only authorized researchers could access. The information was examined using aggregated and anonymized statistics, which did not expose any particular identities. The study's findings were presented in a way that did not reveal any personally identifying information about the participants.

Statistical analysis

Continuous variables were expressed as Mean ±SD. Categorical variables were presented as n (%). The difference between the pre-operative and post-operative periods was determined using the McNemar Test. Sensitivity was calculated by measuring the proportion of actual positives in the total sample and formulated as true positives/(true positive + false negative). The agreement between Lever Test and MRI findings, as determined by Cohen's Kappa coefficient. All calculations were performed with SPSS version 26.0.

## Results

**Figure 1:**
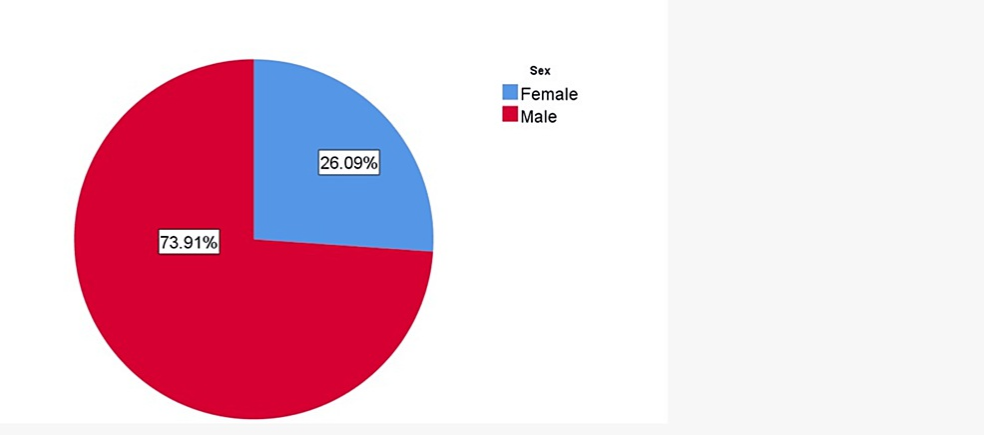
Sex distribution of participants (in %)

The patients were 17 men and six women, with a mean age of 30.4±9.95 years (Figure 1). There were 18 patients with complete tears, four with partial tears, and one with intact ACL damage. Also, in this study, 10 (44%) patients complained in the right knee, while 13 (56%) complained in the left knee. In addition, there were only two (9%) patients who had a generalized ligamentous laxity. In terms of symptoms, 21 (91%) patients complained of giving away, 22 (96%) patients complained of knee pain, and 10 (43%) patients complained of locking of the knee.

Table 1 illustrates that on the ipsilateral leg examination, the pre-operative positivity of Lever Sign was 44 %, Lachman 83 %, and Anterior Drawer 87%. The pre-operative negativity of the Lever Sign was 56%, Lachman 7%, and Anterior Drawer 13%. After giving anesthesia, test positivity rates are 44% for Lever Sign, and 56% for Pivot Shift, and the negativity of the tests after giving anesthesia was 56% for Lever Sign and 44% for Pivot Shift. Post-operative positivity of Lever Sign was 17%, Lachman 39%, and Anterior Drawer 35%, and the negativity of Lever Sign was 83%, Lachman 61%, and Anterior Drawer 65%. Mcnemar Test p values were significant for the difference of positivity anterior drawer test (p=0.002) and were not significant on Lever Sign (p=0.7) and Lachman tests (p=0.13). The sensitivity values of the Lever Sign are 18-41%, Lachman 41%-82%, Anterior Drawer 36-86%, and Pivot Shift 59%.

**Table 1:** Frequencies (N) and percentages (%) of different tests in different situations and it is sensitivity using ipsilateral leg

	Preoperative			Under Analgesia		Postoperative		
	Lever Sign	Lachman Test	Anterior Drawer Test	Pivot Test	Lever Sign	Anterior Drawer Test	Lever Sign	Lachman Test
Positive	10 (44%)	19 (83%)	20 (87%)	13 (56%)	10 (44%)	8 (35%)	4 (17%)	9 (39%)
Negative	13 (56%)	4 (7%)	3 (13%)	10 (44%)	13 (56%)	15 (65%)	19 (83%)	14 (61%)
Sensitivity	41 %	82%	86%	59%	41%	36%	18%	41%
Total	23			23		23		

Correlation analysis was done and show a weak but not statistically significant interrater reliability between MRI findings and Lever sign (p=0.846) (Kappa=0.2)

On the contralateral leg examination, the pre-operative positivity of Lever Sign was 9%, Lachman 17 %, and Anterior Drawer 22%. The pre-operative negativity of the Lever Sign was 91%, Lachman 83%, Anterior Drawer 78% (Table 2).

**Table 2:** Frequencies (N) and percentages (%) of different tests in different situations using the contralateral leg

	Preoperative contralateral leg examination		
	Lever Sign	Lachman Test	Anterior Drawer Test
Positive	2 (9%)	4 (17%)	5 (22%)
Negative	21 (91%)	19 (83%)	18 (78%)
Total	23	23	23

## Discussion

The ACL is one of the most often impaired ligaments in the knee, particularly in athletes who compete in sports that require turning, cutting, or landing from leaps [4]. ACL rupture diagnosis is critical for establishing the best therapy and prognosis for the injury, as well as preventing future damage to the knee joint and related tissues. However, diagnosing ACL rupture can be difficult, especially in the acute phase, when pain, edema, and muscle guarding can make clinical testing difficult to conduct and interpret [5].

Several clinical tests, including the Lachman Test, Anterior Drawer Test, and Pivot Shift test, have been developed and validated for detecting ACL rupture. These tests are based on measuring the tibia's anterior translation relative to the femur, which increases when the ACL is ruptured. These tests, however, have notable drawbacks, including limited sensitivity for partial tears, low specificity for chronic tears, and low interobserver reliability [4]. Furthermore, these tests may cause discomfort or agony to the patient, particularly during the acute period, and may necessitate anesthetic or sedation [6].

Lelli et al. suggested the Lever Sign Test, sometimes known as Lelli's Test, as a novel clinical diagnostic for identifying ACL rupture [3]. Applying a downward force across the distal third of the quadriceps muscle while placing a fist beneath the proximal third of the calf of a supine patient with extended legs is the basis for this test. The reasoning for this test is that when the ACL is intact, the downward force causes an upward shift of the heel due to the ACL's lever action. When the ACL is torn, however, the downward impact causes an anterior translation of the tibia with no upward movement of the heel [2].

Our study suggests that the Lever Sign Test has lower sensitivity than the Lachman Test and Anterior Drawer Test in both pre-operative and post-operative states. Also, the Lever Sign Test has lower sensitivity than the Pivot Shift Test in the under-anesthesia state. Out of all four tests, in both pre-, under-, and post-anesthesia testing, the Lever Sign Test has the lowest sensitivity. Moreover, our study revealed a weak agreement between MRI findings and Lever Sign Test results.

One possible explanation for these findings is that the Lever Sign Test may be more influenced by factors other than ACL integrity, such as quadriceps muscle strength and tone, patellar tendon length and elasticity, tibial tuberosity position and shape, and knee joint effusion [4,7]. These factors may affect the amount of downward force applied to the quadriceps muscle and the degree of upward movement of the heel. Therefore, some patients with intact ACL may have a positive Lever Sign Test due to these factors, while some patients with ruptured ACL may have a negative Lever Sign Test due to these factors.

Another possible explanation is that the Lever Sign Test may not be able to detect partial or chronic ACL ruptures as well as other clinical tests. Partial ACL ruptures may still provide some degree of stability to the knee joint and prevent excessive anterior translation of the tibia [4]. Chronic ACL ruptures may be compensated by other structures such as secondary stabilizers or scar tissue formation [4]. Therefore, some patients with partial or chronic ACL ruptures may have a negative Lever Sign Test due to these reasons.

In addition to our study, other studies have also evaluated the performance of the Lever Sign Test in different settings and populations. Some of these studies are summarized below:

A prospective study by Jarbo et al. found that the Lever Sign Test was positive in 87 of 100 patients (87%) who underwent arthroscopic surgery for ACL injury [8]. The study also reported that the Lever Sign Test had good interrater reliability (Kappa=0.97) and was simple to use.

A cross-sectional study by Rolvien et al. found that the Lever Sign Test had lower sensitivity (85.57%) and specificity (25%) than other clinical tests in a sample of 100 patients with suspected ACL injury [6]. The study also found that the Lever Sign Test had no significant correlation with MRI findings (Kappa=0.11).

A cross-sectional study by Parikh et al. found that the Lever Sign Test was affected by hamstring muscle tension (p<0.001), posterior cruciate ligament (PCL) integrity (p<0.001), meniscal injury (p<0.001), and BMI (p<0.001) in a sample of 100 patients with suspected ACL injury [10].

These studies suggest that the Lever Sign Test may have variable accuracy and reliability depending on various factors such as surgical status, MRI confirmation, hamstring tension, PCL integrity, meniscal injury, and BMI.

Therefore, while the Lever Sign Test appears to be a promising clinical test for identifying ACL rupture, particularly in the acute phase and for full rips, further research is needed to prove its validity and reliability in diverse situations and populations. Furthermore, rather than depending on a single clinical test, it is advised to employ a mix of clinical tests, as well as examine other aspects such as history, mechanism of injury, physical examination findings, and imaging data [4]. This way, clinicians can improve their diagnostic accuracy and provide better care for patients with ACL injuries.

To conclude this discussion, we have reviewed the Lever Sign Test as a novel clinical diagnostic for identifying ACL rupture. We have compared its sensitivity with other established clinical tests such as Lachman Test, Anterior Drawer Test, and Pivot Shift Test. We have also discussed some possible explanations for why the Lever Sign Test may have lower sensitivity than expected. We have also added some data from other studies that have evaluated the performance of the Lever Sign Test in different settings and populations. We have suggested that further research is needed to validate and refine this test in different settings and populations. We have also recommended that clinicians use a combination of clinical tests and other sources of information to diagnose ACL rupture more accurately.

Limitations

This study has several limitations that should be acknowledged. First, the sample size was small and may not be representative of the general population of patients with suspected ACL injuries. Second, the Lever Test was performed by different examiners who may have different levels of experience and skill in performing the test. Third, the MRI findings were not confirmed by arthroscopy, which is the gold standard for diagnosing ACL injuries. Fourth, the Lever Test was only performed in the supine position, which may not reflect the functional state of the knee during daily activities. Fifth, the study did not compare the Lever Test with other novel clinical tests, such as the Lateral Pivot Shift Test or the ACL Suture Device Test. Therefore, further studies with larger sample sizes, standardized examiners, arthroscopic confirmation, and comparison with other clinical tests are needed to validate the accuracy and reliability of the Lever Test.

## Conclusions

In conclusion, our findings show that the Lever Sign Test cannot be used alone to rule out ACL injuries in individuals with acute knee pain and instability. In addition, the test should not be used in isolation, but rather as an addition to other clinical tests with better sensitivity and reliability, such as the Lachman Test, Anterior Drawer Test, and the Pivot Shift Test. Furthermore, our findings suggest that the Lever Sign Test has lower accuracy than other clinical tests when comparing the results of tests with MRI findings, which is the most commonly used imaging modality for diagnosing ACL injuries. Therefore, the Lever Sign Test should be used with caution and only as a supplementary tool for assessing ACL integrity. The Lever Sign Test may have some advantages over other clinical tests, such as being easy to perform, requiring minimal equipment, and being less affected by anesthesia. However, these advantages are outweighed by the low sensitivity and specificity of the test, especially in cases of partial tears or chronic injuries. The Lever Sign Test may also have some limitations in terms of interrater reliability, as different examiners may have different levels of experience and skill in performing the test. Moreover, the Lever Sign Test was only performed in the supine position, which may not reflect the functional state of the knee during daily activities. Therefore, further studies with larger sample sizes, standardized examiners, arthroscopic confirmation, and comparison with other novel clinical tests are needed to validate the accuracy and reliability of the Lever Sign Test.
